# Bonding of Core Build-Up Composites with Glass Fiber-Reinforced Posts

**DOI:** 10.3390/dj7040105

**Published:** 2019-11-05

**Authors:** Margarita Fragkouli, Ioannis Tzoutzas, George Eliades

**Affiliations:** 1 Department of Operative Dentistry, School of Dentistry, National and Kapodistrian University of Athens, 11527 Athens, Greece; ioannistzoutzas@gmail.com; 2 Department of Biomaterials, School of Dentistry, National and Kapodistrian University of Athens, 11527 Athens, Greece; geliad@dent.uoa.gr

**Keywords:** FRC posts, core build-up composites, bond strength, failure mode

## Abstract

The purpose of this study was to investigate the bonding capacity of composite core build-up materials with prefabricated glass fiber-reinforced posts possessing different coronal morphologies. Five post types (Archimede Line (ARL), Fibrekleer (FBK), Glassix (GLX), Matrix Plus (MTP), and ParaPost White (PRW) and three core build-up materials (ClearfilPhoto Core (CPC), ClearfilDC Core (CDC), ClearfilNew Bond (CNB) of different curing modes (light-, self-, dual-cured respectively) were selected. The coronal part was embedded in the core build-up materials and the specimens were loaded under tensile force up to failure. The reliability (β) and characteristic life (σο, in Ν) of the debonding force were evaluated by Weibull statistics and the debonded specimens were subjected to failure mode analysis. The results showed that ARL, MPT posts were the most and GLX the least retentive, despite the core build-up material used. CPC provided the highest retention with four posts (FBK, GLX, MTP, and PRW), without statistically significant differences from CDC in two (FBK and MTP) and CNB in one (PRW). CPC and CDC were the most reliable core materials for two posts (ARL and PRW), with no statistically significant difference from CNB in three (FBK, GLX, and MTP). GLX and PRW demonstrated the highest (93%) incidence of post detachment from core, whereas FBK demonstrated the highest percentage of core material fracture, with most fractures occurring in CDC (57%). Post fractures were most prominent in MTP when combined with CNB. The presence of specific coronal retentive features did not essentially ensure increased strength with the core material, due to their delamination.

## 1. Introduction

The restoration of root canal-treated teeth still remains a challenging issue regarding functionality, durability and aesthetics. Since these teeth exhibit a higher fracture risk from vital teeth, they frequently require post restoration [1,2,3]. The development of aesthetic ceramic and polymer materials with enhanced mechanical properties led to the design of a variety of non-metallic posts including aesthetic glass fiber-reinforced composite (GFRC) posts [4,5]. These posts contain different glass fibers, embedded in highly crosslinked polymer structures [6,7,8]. The glass fibers of GFRC posts are made of electrical glass (e-glass: SiO_2_, CaO, B_2_O_3_ and alkali metal oxides), which is the most common type of glass used, or of high-strength glass (s-glass: SiO_2_, Al_2_O_3_ and MgO) [9]. The important characteristics of the fibers are the type, length, diameter, direction, density and surface treatment, for adequate bonding with the polymer matrix [10,11,12]. The matrix is composed of epoxy, Bisphenol A diglycidylmethacrylate (Bis-GMA)-type or, more rarely, of high-molecular-weight polymethyl methacrylate (PMMA) polymers [8,13]. In addition to the glass fibers, it may contain particulate fillers for strength and radiopacity. The glass fiber component provides rigidity and increased tensile strength, whereas the resin matrix holds the fibers in place and absorbs the functional stresses applied throughout the post system [14], which are developed at the fiber–matrix interface and extend along the fiber surface [15]. Unfortunately, there is limited information for the mechanical properties of the resin matrices used, which contribute, as well, to the post strength [12,16]. Another important factor is the presence of coupling agents at the fiber–matrix interface, since this may affect the fatigue strength and structural integrity of the post [12,16]. Finally, structural imperfections such as voids and microcracks developed during the manufacturing process and weaken the post [12].

The greatest advantage of the GFRC posts is their modulus of elasticity, which ranges between 18 and 20 GPa, resembling that of dentin [17,18,19]. Assuming a durable bond between the composite core build-up material, post and dentin, the entire restoration acts as a homogeneous biomechanical unit [20], reducing the risk of root fracture, microleakage, secondary caries and re-infection of the periapical area [21,22,23,24]. The flexural strength of these posts is equally important, since a very flexible post can lead to restoration fracture [16]. In GFRC posts, the mechanical properties depend on the direction of the applied force and the material structure due to their anisotropic nature. Consequently, the failure of GFRC is caused by different mechanisms, such as matrix cracking, fiber fracture and interfacial detachment [25].

Bonding of the GFRC posts to root dentin has been the subject of many studies. Various intracanal post designs, conditioning/priming treatments of post and intracanal walls and types of luting agents have been tested to establish a strong and durable post bonding by combining mechanical retention and chemical adhesion principles [26,27,28,29]. Besides, adhesive techniques have been introduced for the treatment of the coronal part of the post prior to the application of the core build-up composite restoration. Light-, self- or dual-cured composite restorative materials of high, medium or low viscosity are used for post-retained core build-up restorations in root canal-treated teeth [2,30]. The post surface treatment, the type of the composite material used, and the coronal post morphology have been identified as the main variables affecting the strength [26,27,31,32]. However, the documentation for the effect of the latter is limited.

The purpose of this study was to investigate the factors affecting the bonding of different composite core build-up materials with the coronal part of GFRC posts. The null hypotheses tested were: (1) the morphology of the coronal surface of the post does not affect the bond strength with the restorative material, and (2) the type of the restorative material used (light-, self- or self-cured) does not affect the bond strength with the post.

## 2. Materials and Methods

**Figure 1:**
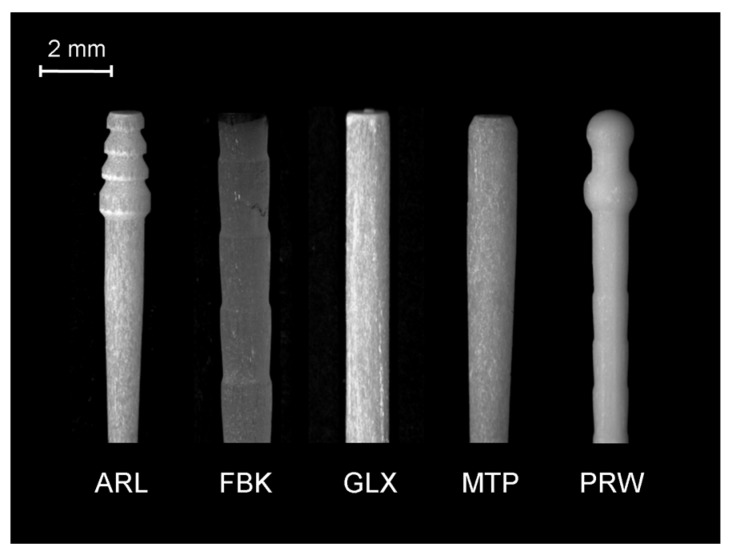
Stereomicroscopic image of the coronal and middle body parts of the posts used in the study (10×, bar = 2 mm).

Five types of GFRC posts (Figure 1) and three composite core build-up materials of different curing modes (light-, self-, dual-cured) were selected for the study (Table 1).

**Figure 2:**
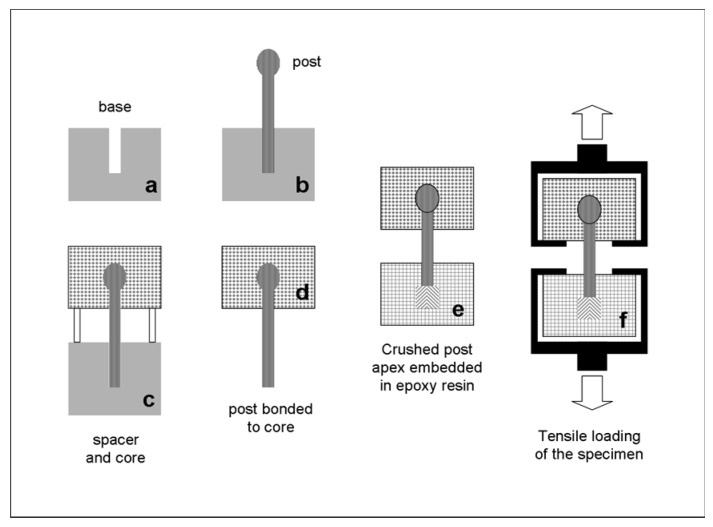
The specimen preparation procedure employed.

The posts selected represented the majority of the coronal designs currently available. ARL had three overlapping conical retentive elements at the coronal part, a smooth parallel-sided body and a tapered middle, apical part. Fibrekleer (FBK) was parallel with serrations. Glassix (GLX) was smooth parallel, with a small tapered length at the apex. Matrix Plus (MTP) was smooth parallel, with a middle-apical taper. ParaPost Fiber White (PRW) had two interconnected spheres as coronal retentive elements and a parallel body with serrations. The serrated posts exhibited rounded reverse buttress thread edges of a small angle relative to the post length and small thread depths. A silane with a phosphate monomer (Monobond Plus, Ivoclar Vivadent, Schaan, Liechtenstein) was used for post priming prior to composite application. After pilot experiments, 20 samples were prepared per post and core build-up material. The specimen preparation was as follows (Figure 2).

Canal lengths of 12 mm were made at the central part of high-molecular-weight polyethylene bases using drills corresponding to the post sizes selected (Figure 2a) and the posts were seated after silanization without cementation in the drilled canals, with 4 mm of the post head extending from the base surface (Figure 2b). On these bases, transparent polycarbonate ring spacers and molds were placed and filled with the corresponding composite core build-up material (5 mm in height, 9 mm in internal diameter, Figure 2c). The light- and dual-cured materials were irradiated with an LED curing unit (Demi Plus Light Curing System, Kerr Hawe, Middleton, WI, USA, light intensity varying between 1.100 and 1.300 mW/cm^2^) for 10 s from three lateral surfaces, at 120° angle directions each. Then, the posts with the cores were removed from the canals (Figure 2d), their apices were pressed with a metal pliers up to a 2 mm length from the apical tip to separate the fibers for increased mechanical retention, and they embedded in epoxy resin up to a 5 mm length from the tip (Figure 2e). The samples were stored for 48 h under dark and dry conditions before testing. Custom-made self-aligning stainless steel grips were used to load each specimen under tensile force up to failure in a universal testing machine (Imperial 2500, Mecmesin, Slinfold, West Sussex, UK), operating at 10 mm/min cross-head speed and 100 Hz sampling rate (Figure 2f). The debonded specimens were examined under a stereomicroscope (M80, Leica Microsystems, Wetzlar, Germany) to evaluate the failure mode. The types of failures were classified as type I (post detachment from the core), type II (core fracture with intact post), type III (post fracture at the free middle portion) and type IV (post fracture into the epoxy resin). Selected specimens were further examined by a scanning electron microscope (Quanta 200, FEI, Hilsboro, OR, USA) operated in the low vacuum (LV) mode (20 kV, 90 μA, 133 Pa, solid state backscattered detector-BE).

Descriptive statistics were used to report the results. For statistical comparisons among posts and core materials, Weibull analysis was employed. The reliability (β-shape parameter), fracture probability or characteristic life (σο-scale parameter), correlation coefficient (r^2^) and 95% confidence intervals (CIs) were calculated and compared. The statistical analysis was performed using the OriginLab software (v.9.1 SRO, Northampton, MA, USA). For all cases, a 95% confidence level was used.

## 3. Results

**Figure 3:**
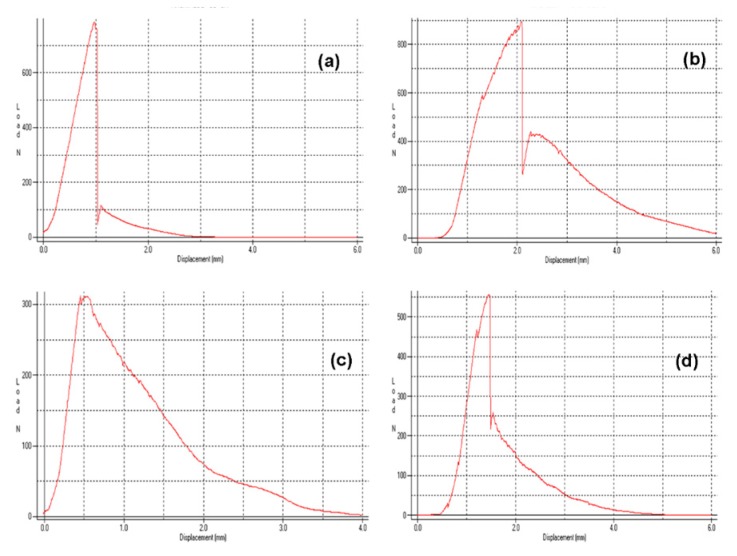
Load–displacement curves of ARL (**a**), FBK (**b**), GLX (**c**) and MTP (**d**). Note the stepwise loading in FBK after initial fracture.

**Figure 4:**
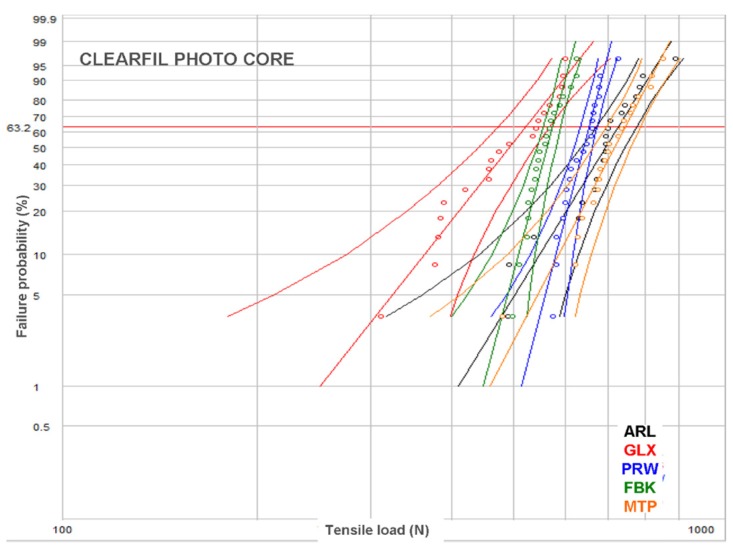
Weibull plot for the posts restored with CPC.

**Figure 5:**
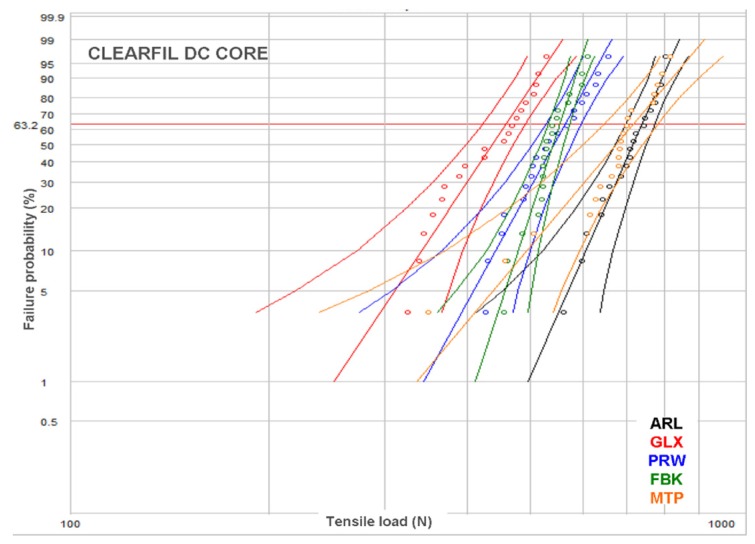
Weibull plot for the posts restored with CDC.

**Figure 6:**
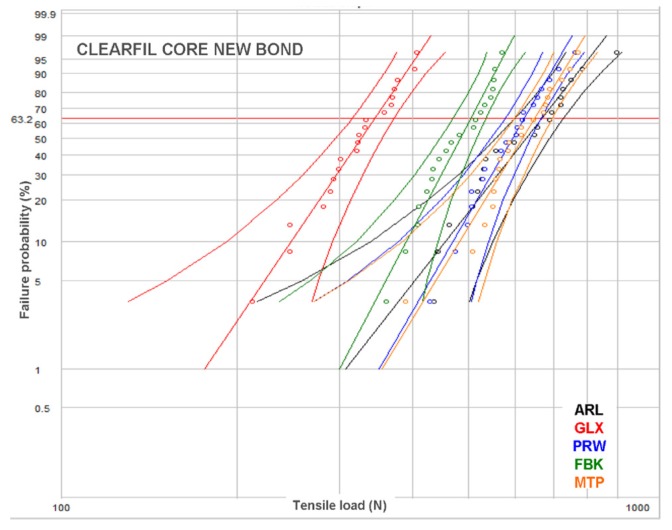
Weibull plot for the posts restored with CNB.

Representative load–displacement curves are illustrated in Figure 3. All curves demonstrated an initial peak, considered as the breaking point, with a secondary lower-slope loading prior to complete debonding. For smooth posts, the secondary loading phase was negligible. However, a small stepwise secondary loading pattern was observed in posts with retentive features in their main body, rather than the coronal part. The results of descriptive statistics for the post–core pairs are presented in Table 2. The Weibull graphs for the posts per core material are depicted in Figure 4, Figure 5 and Figure 6. The reliability, failure probability and correlation coefficient of the post–core pairs are presented in Table 3.

The post reliability (β) per core build-up material showed statistically significant differences only in CPC, with the ranking FBK, PRW > ARL, GLX. MTP demonstrated insignificant differences from both these statistically homogeneous groups. For the reliability of core materials per post, statistically significant differences were found only in ARL (CDC > CNB, with CPC manifesting no significant difference from each one) and PRW (CPC > CDC > CNB). The statistical ranking of the characteristic life (σο) of posts per core build-up material were MTP, ARL > PRW > FBK > GLX for CPC, ARL, MPT > PRW, FBK > GLX for CDC and ARL, MPT > MTP, FBK > GLX for CNB, with PRW exhibiting significant higher values only from GLX. The characteristic life of core materials per post were CPC, CDC > CNB for FBK, CPC > CDC > CNB for GLX, CPC > CNB, with CDC showing no differences from each one for MTP, and CPC > CDC, with CNB showing no differences from each one for PRW. No differences were registered for any of the core materials in ARL.

**Figure 7:**
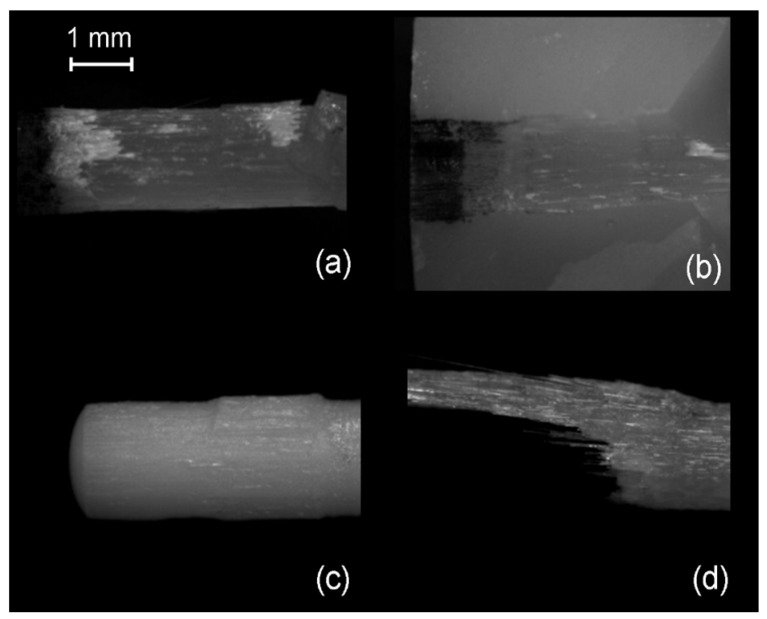
Stereomicroscopic images of fractured posts and core build-up materials (25×, bar = 1 mm). (**a**) FBK coronal part with broken serrations; (**b**) FBK broken part into the core material; (**c**) PRW with sheared off spherical retentive elements; (**d**) ARL body fracture.

Representative stereomicroscopic images of the debonded posts are illustrated in Figure 7a–d including fracture of the superficial post structure with retention of fibers into the broken part of the composite core (Figure 7a,b), destruction of the post coronal retentive features (Figure 7c) and cohesive post failures, with separation of the fibers from the matrix (Figure 7d).

**Figure 8:**
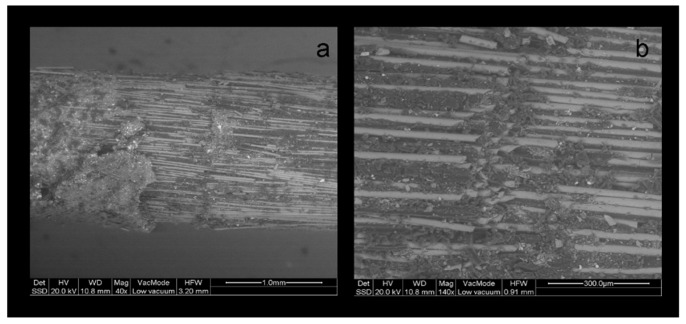
BE LV-SEM image of a fractured PRW post, (**a**) with details of the sheared off spherical protrusions (**b**) and the cut fibers at the contact of the protrusions with the main body (a: 40×, bar = 1 mm, b: 140×, bar = 300 μm).

**Figure 9:**
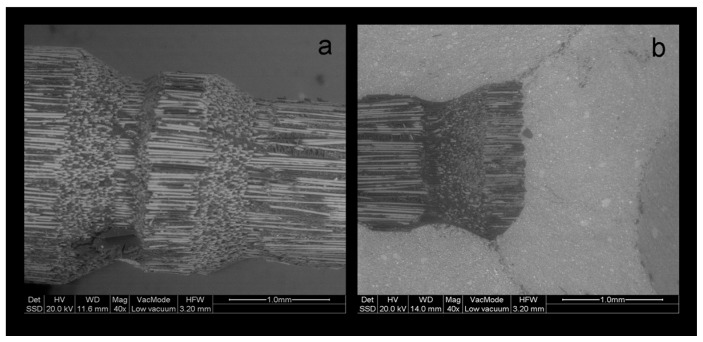
BE LV-SEM image of a debonded ARL post, (**a**) with sheared off retentive elements and (**b**) attached to the core material structure (a: 40×, bar = 1 mm, b: 140×, bar = 300 μm).

In several cases, the LV-SEM examination demonstrated post debonding, with fracture of the superficial fiber layer which was attached onto the broken part of the core composite resembling an impression of the affected zone (Figure 8 and Figure 9).

**Figure 10:**
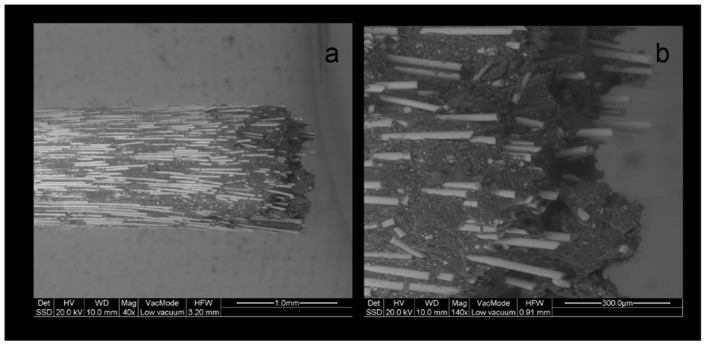
BE LV-SEM image of a fractured MTL post, (**a**) with details (**b**) of non-uniformly distributed fibers (a: 40×, bar = 1 mm, b: 140×, bar = 300 μm).

In the post images, it was apparent that the specific coronal features were produced by breaking off the longitudinally packed fibers accordingly, creating structural discontinuities. The complete post fractures showed protruding fibers from a distorted and broken matrix (Figure 10).

The results of the failure mode analysis are summarized in Table 4.

GLX and PRW demonstrated the highest (93%) and MTP and FBK the lowest (5–10%) incidence of post detachment from core (type I failure), irrespective of the composite build-up material used. The highest overall percentage of core material fracture (type II failure) was observed in FBK (37%), followed by MTP (22%), ARL, PRW (7% each) and GLX (no fracture). Most core material fractures occurred in CDC (57%), less in CNB (11%) and the least in CPC (6%). Post fractures at the free middle portion (type III failures) were most prominent in MTP (17%), followed by GLX (7%), ARL (4%) and the group of FBK and PRW (no fractures). CNB was the core material with the highest (10%) type III failures, CPC exhibited 6% and CDC only 1%. No post apex fractures into the epoxy resin (type IV failure) were documented for GLX and PRW. However, this type of failure was observed in ARL (58%), MTP (56%) and FBK (53%) irrespective of the core material used. The ranking of type IV failures within the core materials groups was CPC (47%), CNB (38%) and CDC (16%).

## 4. Discussion

Since the introduction of GFRC posts for the restoration of root canal-treated teeth, the main issue raised was the optimal bonding to the root canal walls [33,34,35,36] because predictable resin adhesion can be achieved mainly with the cervical part of root canal dentin [37]. However, the resistance to the detachment of the composite core build-up material from the post is equally important, since it is recognized as the weak link in the bonding process in many studies [26,38]. Currently, new posts are available with coronal morphology favoring mechanical retention and the chemical adhesion of the composite core build-up materials, resulting in a greater mechanical strength than the posts with a plain cylindrical coronal part [12].

The present study was undertaken aiming to assess the contribution of the post coronal design to the bonding capacity with three types of core build-up particle-filled composite materials (light-, self- and dual-cured), all routinely used in clinical practice. The push-out tests usually performed to evaluate the bond strength of smooth post–core interfaces [39] cannot be used in cases of complex coronal morphology. For this reason, a more relevant tensile test was employed. The key point in the experimental setup used was to establish the most efficient way of post apex retention with the epoxy resin, as the coronal features of many posts provided higher core retention, leading to post apex detachment. Crushing the fiber binder at the apical 2 mm length facilitated strong retention of the post, because the contact of individual fibers with the embedding material was greatly increased. The load–displacement curves showed repeatability for the posts tested, establishing the credibility of the method used. A secondary post-fracture stepwise loading pattern, with multiple small peaks was observed in posts with universal coronal and body retention elements (i.e., FBK). These overlapping load–displacement curves with minimal stress relaxation create greater cumulative displacement values, which may delay post body detachment/fracture in comparison with smooth morphologies. The post body designs may affect the retention and stress distribution patterns at the interfaces, as the part of the body was included in the core material; the cylindrical posts create more shear stresses, the tapered more compressive, whereas the serrated a balance of shear and compressive forces in favor of the former, due to the small angulation of the buttress-thread edges.

In the present study, a Weibull statistical analysis was preferred over a typical regression analysis, since bond strength tests usually demonstrate a brittle fracture behavior, by measuring the stress to initiate failure from an existing defect, creating thus data scattering and reproducibility problems. Expressing the results in fracture probability at a particular stress by Weibull statistics, has long been recognized as a better method for interpretation of bond strength data [40]. Nevertheless, to fulfill the purpose of direct comparisons with other studies, the results of the descriptive statistics have been included as well. The strength values were given in force (N) and not in stress (MPa) units, since the loaded area was very complex including the different morphologies of the core-embedded coronal, the epoxy-embedded apical and the free intermediate post parts. The most reliable posts, in terms of data reproducibility (β), were the FBK and PRW and the least GLX and MTP, mainly based on the differences encountered in CPC. This implies that, for the current experimental design, serrated posts were the most reliable, irrespective of the presence of specific retentive elements. For core materials, the light-cured CPC was the most reliable, whereas the self-cured CNB was the least. This ranking may reflect the advantages of the former, such as the higher C = C conversion, higher setting shrinkage rate adding residual compressive stresses at the post–core interface and less porosity, as no mixing is required [41]. Regarding the post–core strength (σο), MTP and ARL demonstrated the highest and GLX the lowest values, independently of the core materials used. It is interesting that the highest values were experienced with smooth shape posts with or without coronal retention features, while another post of the same category without retention features was ranked as the weakest. CPC was identified as the core material providing the highest σo in four posts, but with insignificant differences from CDC in MTL and CNB in PRW, whereas CNB was ranked last in four out of five posts. However, the complexity of the loading pattern by combining different post morphologies and core material properties does not allow for a sound conclusion, without considering the failure mode. The greatest percentage of type I and II (post debonding, core fracture) and the lowest of type III and IV failures (post fracture at main body and embedded apex) in the CDC group imply a weaker core material, possibly attributed to the lower inorganic filler content (52 vol % vs. 68 and 63 vol % for CPC and CNB respectively) of the same resin matrix, apparently to achieve the flow consistency. Flowable composites may demonstrate better adaptation to complex post morphologies due to the lower viscosity, increased friction fitting appended to their higher setting shrinkage and a porous-free structure attained by the automix cartridge delivery systems. Nevertheless, the greater elasticity and lower cohesive strength of these materials may affect the post–core system strength [15,42]. In ARL, with highest σο values, CDC showed the highest percentage of type I failures as a result of the lowest mechanical properties of the core material and the fracture of the retentive cones. This finding was even more pronounced in PRW, with medium ranked σο values, highest type I failures (93% average) and no difference between the core materials tested. The protruding coronal spherical retentive elements, possessing lower mechanical retention capacity from the conical of ARL, were destroyed after loading by the shear force components developed at the region (Figure 7c). The low percentage of fibers (per weight and cross-sectional area) documented for this post may explain this behavior [15,43]. The cylindrical design of the smooth GLX post, with the small apical taper, was the least retentive with the highest percentage of type I failures (93% average). The limited mechanical retention and the greatest contribution of the shear forces at the core–post interface may corroborate these results. However, the smooth and tapered post MTP provided high strength and a high post fracture incidence (type III, IV failures) of 50–95% (average 73%), with most failures being of type IV (average 56%). An explanation for these findings may be given based (a) on the greater MTP surface area in contact with the core material, due to the greatest diameter of the coronal part and (b) on differences in the roughness parameters of the two types of macroscopically smooth surfaces. A serrated post (FBK) demonstrated higher strength next to GLX, but inferior to all other posts, with a 53% post apex fracture and the highest type II failure mode (average 37%). The design of these posts is known to enhance retention in comparison with smooth posts, but it reduces the post rigidity due to fiber discontinuity in the serrated part [2]. This design though, induced high internal stress concentration in the core material creating catastrophic failures, especially when used in combination with the weaker, low particle-filled CDC flowable composite. The highest percentage of type IV failures in the high strength MTP and ARL posts (56% and 58% respectively) may be associated with the taper design of their apex, which provides higher stress concentrations due to the reduced post diameter.

The core materials used do not possess any self-adhesive properties with the posts, but they contribute to their frictional retention by wetting and polymerization shrinkage [44,45,46]. To improve the interfacial strength of intracanal luting, silanization has been advised and documented [47,48,49], but the effect of silanization on the strength of the coronal restoration is not fully documented yet [15,42,43,46,47,50] although the same mechanisms may apply. In the present study, a silane primer was used containing γ-methacryloxypropyl trimethoxysilane (γ-MPTS), the silane used in most dental applications, along with 10-methacryloyloxy dihydrogen phosphate (10-MDP), a well-established phosphate monomer with bonding capacity to ceramic oxides. The combination of these two monomers has been considered to offer synergy in bonding to diverse substrates [48], although doubts have been expressed on the reactivity of the silanes in such products [48,49,51,52]. This primer has an acidic pH, which may exert an inhibitory effect on slow setting self-cure materials, like CBN, by inducing amine protonization [51,52,53,54]. Such reactions lead to inadequate interfacial curing [44]. The extent up to which this mechanism is implicated in the lowest σo values experienced with CNB in three out of the five posts used is unknown. This mechanism may not apply to the dual-cured CDC. Although this material has a rather weak self-curing catalyst component, it was thoroughly light-cured. Similar were the results for the light-cured CPC; the fast setting reaction of the light-cured materials does not allow diffusion of the acidic primer into the core and pronounced deactivation of the amine component of the amine-benzoyl peroxide redox system [53,54].

Summarizing the results of the present study, the first null hypothesis should be rejected, since the presence of specific retentive elements at the coronal part of the posts did not affect the bond strength with the core build-up material. The second hypothesis should be partially accepted, since, in four out of the five posts, the heavily filled light-cured material demonstrated the highest retention values and the more favorable failure mode. The clinical significance of these results should be interpreted with caution. To simplify the loading pattern and facilitate the tensile experiment, most of the post length was left free. Therefore, the fracture strength measured mainly reflects the retention capacity of the core material and the tensile strength of the post. This is not the case in clinical practice where the entire intracanal post body is bonded to dentin with resin cements or flowable core materials. However, such a simulation does not allow for assessment of the core–post strength at the coronal part, as flowables are much weaker than the paste core materials used in the study. No means of aging was performed, although it is accepted that the GFRC posts are affected by changes in humidity and temperature [16], since direct post exposure to such environments is limited. Further studies are required to evaluate the fatigue factors affecting retention, efficacy, and durability of core–GFRC post systems, to verify the best combination.

## 5. Conclusions

Under the experimental conditions of the present study, the following conclusions can be reached:

1. The coronal parts of ARL and MPT posts were the most retentive, whereas GLX was the least retentive, irrespective of the core build-up material used.
2. CPC provided the highest retention with four posts (FBK, GLX, MTP, and PRW), without statistically significant differences from CDC in two (FBK and MTP) and CNB in one (PRW). CPC and CDC were the most reliable core materials for two posts (ARL and PRW), with no statistically significant difference from CNB in three (FKB, GLX, and MTP).
3. The presence of specific coronal retention features in some posts (ARL and PRW) did not essentially ensure increased strength, due to shear fracture of the retentive features.

## Figures and Tables

**Table 1:** The materials used in the study.

Product (Code)	Composition *	Manufacturer
**A. Posts**		
Archimede Line (ARL)	Glass fiber, composite (diameter: 1.2 mm)	Innotech, Robbio, Italy
Fibrekleer (FBK)	Bis-EMA, HDDMA, glass oxide 30–60%, YbF_3_ 10–30 wt % (diameter: 1.5 mm)	Pentron, Wallingford, CT, USA
Glassix (GLX)	Epoxy resin, braded glass fiber (65 wt %, diameter: 1.5 mm)	R. Nordin, Montreux, Switzerland
Matrix Plus (MTP)	Glass fiber, composite (diameter: 2.0 mm coronal, 1.2 mm apical)	Innotech, Robbio, Italy
ParaPost Fiber White (PRW)	Resin 29%, glass fiber 42%, fillers 29 wt % (diameter: 1.5 mm)	Coltene/Whalede nt, Mawhaw, NJ, USA
**B. Core Build-up Composites**		
Clearfil Photocore (CPC)	Bis-GMA, TEGDMA, silanated silica, silanated barium glass (total filler: 83 wt %, 68 vol %), camphoroquinone, accelerators	Kuraray Medical Inc, Okayama, Japan
Clearfil DC Core (CDC)	Bis-GMA, TEGDMA, hydrophilic aliphatic dimethacrylate, hydrophobic aromatic dimethacrylate, silanated barium glass, silanated colloidal silica, colloidal silica, alumina (total filler: 74 wt %, 52 vol %), camphoroquinone, accelerators.	Kuraray Medical Inc, Okayama, Japan
Clearfil Core New Bond (CNB)	Bis-GMA, TEGDMA, silanated silica, colloidal silica, silanated lanthanum glass (total filler content: 78 wt %, 63 vol %), benzoyl peroxide-amine	Kuraray Medical Inc, Okayama, Japan

* According to the manufacturers’ information. Bis-EMA: Ethoxylated bisphenol-A dimethacrylate, HDDMA: 1,6-hexanodiol dimethacrylate, Bis-GMA: Bisphenol A diglycidylmethacrylate, and TEGDMA: Triethylene glycol dimethacrylate.

**Table 2:** Results of descriptive statistics for the fracture strength (in N) of the post–core pairs tested.

Descriptive Statistics (n = 20/post)	Core	Post				
		ARL	FBK	GLX	MTP	PRW
Mean standard deviation 95% CI of mean median 5–75% percentiles	CPC	684.9	559	486.3	707.1	637.7
		97.7	37.1	88	83.7	41
		45.7	17.4	41.2	39.2	19.2
		694	553.5	482.7	701	645.7
		652.6–738.7	528.8–583.5	405.1–561.9	669–761.7	602.4–667
Mean standard deviation 95% CI of mean median 25–75% percentiles	CDC	706.5	535.8	431.7	665.9	536.1
		69.7	41.3	66.3	115.3	68.9
		32.6	19.4	31	54	32.2
		712.3	528.7	440.6	684	528.9
		650.9–767.1	520.6–561.2	369–487.8	633.2–739.7	490.2–590.3
Mean standard deviation 95% CI of mean median 75% percentiles	CNB	619.7	478	323.1	611.3	588.4
		126.8	63.4	53.1	91.2	86.2
		59.4	29.7	24.8	42.7	40.3
		622.9	474.1	323.2	600.9	590.7
		521.6–720.3	428.3–538.4	369.2	554.9–679.9	517.4–651.3

**Table 3:** Results of the Weibull analysis for reliability (β), characteristic life (σο) and the correlation coefficient of the measurements (r^2^). Same lower-case letters show insignificant differences for β and σο between the posts bonded to the same core material, whereas same upper-case letters demonstrate insignificant differences between the same post bonded to different core materials (*p* > 0.05).

Core	Weibull Parameter	Post				
		ARL	FBK	GLX	MTP	PRW
CPC	β (95% CI)	8.1 ^a,A,B^ (5.9–11.3)	15.9 ^b,A^ (11.4–21.9)	6.8 ^a,A^ (4.7–9.8)	10.2 ^a,b,A^ (7.3–14.3)	16.7 ^b,A^ (12.1–23)
	σο (N) (95% CI)	725.2 ^a,A^ (685.1–767.6)	576.4 ^b,A^ (559.8–593.6)	521.9 ^c,A^ (487.8–558.4)	741.9 ^a,A^ (709.1–776.4)	656.7 ^d,A^ (638.7–675.3)
	r^2^	0.93	0.92	0.96	0.94	0.91
CDC	Β (95% CI)	12.9 ^a,A^ (9–18.4)	14.4 ^a,A^ (10.3–19.9)	7.9 ^a,A^ (5.6–11.3)	8.1 ^a,A^ (5.6–11.7)	8.9 ^a,B^ (6.3–12.0)
	σο (N) (95% CI)	736.3 ^a,A^ (710.4–763.1)	554.5 ^b,A^ (536.8–572.7)	459.4 ^c,B^ (433.5–486.9)	708.8 ^a,A,B^ (670.1–749.8)	566 ^b,B^ (537.3–596.2)
	r^2^	0.99	0.93	0.94	0.9	0.95
CNB	β (95% CI)	5.4 ^a,B^ (3.9–7.5)	9.1 ^a,A^ (6.4–12.9)	7.3 ^a,A^ (5.1–10.4)	7.9 ^a,A^ (5.6–11)	7.6 ^a,B^ (5.5–10.6)
	σο (N) (95% CI)	670.9 ^a,A^ (615.7–731.1)	505.1 ^b,B^ (480.1–531.4)	344.9 ^c,C^ (323.8–367.4)	649.2 ^a,B^ (612.1–688.5)	625.1 ^a,A,B^ (588.1–664.4)
	r^2^	0.93	0.96	0.98	0.99	0.97

**Table 4:** Results of failure mode analysis.

Type of Failure	Core	Post					Total per Core (n = 100)
		ARL	FBK	GLX	MTP	PRW	
I	**CPC**	6 (30%)	0 (-)	17 (85)	0 (-)	18 (90%)	41
II		0 (-)	3 (15%)	0 (-)	1 (5%)	2 (10%)	6
III		2 (10%)	0 (-)	3 (15%)	1 (5%)	0 (-)	6
IV		12 (60%)	17 (85%)	0 (-)	18 (90%)	0 (-)	47
I	**CDC**	9 (45%)	6 (30%)	20 (100%)	3 (15%)	19 (95%)	57
II		4 (20%)	14 (70%)	0 (-)	7 (35%)	1 (5%)	26
III		1 (5%)	0 (-)	0 (-)	0 (-)	0 (-)	1
IV		6 (30%)	0 (-)	0 (-)	10 (50%)	0 (-)	16
I	**CNB**	3 (15%)	0 (-)	19 (95%)	0 (-)	19 (95%)	41
II		0 (-)	5 (25%)	0 (-)	5 (25%)	1 (5%)	11
III		0 (-)	0 (-)	1 (5%)	9 (45%)	0 (-)	10
IV		17 (85%)	15 (75%)	0 (-)	6 (30%)	0 (-)	38
I	**Total per post** **(n = 60)**	18 (30%)	6 (10%)	56 (93%)	3 (5%)	56 (93%)	
II		4 (7%)	22 (37%)	0 (-)	13 (22%)	4 (7%)	
III		3 (5%)	0 (-)	4 (7%)	10 (17%)	0 (-)	
IV		35 (58%)	32 (53%)	0 (-)	34 (56%)	0 (-)	
